# Electrochemical Machining of Highly Strain-Hardenable High-Entropy FeMnCrCoSi Alloy: Role of Passivation and Selective Dissolution

**DOI:** 10.3390/ma18214881

**Published:** 2025-10-24

**Authors:** Kavindan Balakrishnan, Kundan Kumar, Indrajit Charit, Krishnan S Raja

**Affiliations:** 1 Nuclear Engineering and Industrial Management, University of Idaho, Idaho Falls, ID 83402, USA; bala4814@vandals.uidaho.edu (K.B.); icharit@uidaho.edu (I.C.); 2 Department of Natural Resource and Society, College of Natural Resources, University of Idaho, Idaho Falls, ID 83402, USA; kundank@uidaho.edu

**Keywords:** high-entropy alloy, electrochemical machining, chloride electrolyte, nitrate electrolyte, passivation, X-ray photoelectron spectrometry (XPS), potentiodynamic polarization, Faradaic efficiency, current density

## Abstract

Fe_42_Mn_28_Cr_15_Co_10_Si_5_ is a highly strain-hardenable high-entropy alloy (HEA) that is challenging to machine with traditional metal cutting tools. The electrochemical behavior of this HEA was examined in nitrate- and chloride-based electrolytes to understand the electrochemical machining (ECM) process. Potentiodynamic and potentiostatic tests were conducted on this alloy in 1 M and 2.35 M NaNO_3_ solutions, with and without additions of 0.01 M nitric acid and 0.01 M citric acid. A 20% NaCl solution was also tested as an electrolyte. Nitrate solutions caused passivation of the HEA, while no passivation was observed in chloride solutions. Surface analysis with X-ray photoelectron spectrometry (XPS) indicated that adding citric acid helped reduce surface passivation. The Faradaic efficiency of ECM increased with higher applied voltage. The chloride solution showed higher Faradaic efficiency than nitrate-based solutions. Specifically, the Faradaic efficiency of 20% NaCl at 10 V is 57.4%, compared to 21.9% for 20% NaNO_3_ + 0.01 M citric acid at 10 V. Electrochemical parameters, including anodic and cathodic exchange current densities, Tafel slopes, and corrosion current densities, were calculated from the experimental data. The corrosion current densities in the 20% nitrate solutions ranged from 2.35 to 3.2 × 10^−5^ A/cm^2^, while the 20% chloride solution had a lower corrosion rate at 1.45 × 10^−5^ A/cm^2^. These electrochemical parameters can help predict the dissolution behavior of the HEA in nitrate and chloride solutions and aid in optimizing the ECM process.

## 1. Introduction

High-entropy alloys (HEA), prepared by four or more equimolar additions of alloying elements, exhibit interesting microstructural and mechanical properties due to a combination of several effects, including the high-entropy effect, the severe lattice distortion effect, the sluggish diffusion effect, and the cocktail effect [1,2,3]. In addition to these effects, transformation-induced plasticity (TRIP) and twin-induced plasticity (TWIP) effects are incorporated in the recently developed high-entropy alloys, which show enhanced strength and ductility [4,5,6,7]. Fe_42_Mn_28_Co_10_Cr_15_Si_5_ is a metastable high-entropy alloy that undergoes a strain-induced phase transition from *γ-fcc* to *ε-hcp* [8]. Metastability is related to the stability of the *fcc* matrix during deformation of the high-entropy alloy. The deformation mechanism is influenced by the stacking fault energy (SFE) of the HEA, which in turn is a function of composition and temperature [9]. When the width of the stacking fault is narrow, dislocation slip is the dominant deformation mechanism, which occurs when the SFE exceeds 45 mJ/m^2^. As the SFE decreases, the stacking fault width increases, making slip-induced deformation less favorable, and the mechanism transitions to twin-induced plastic deformation (SFE = 20–45 mJ/m^2^) or transformation-induced plastic deformation (SFE < 15 mJ/m^2^) [10]. A combination of TRIP and TWIP could operate when the SFE ranges between 13 and 18 mJ/m^2^ [11]. Additions of Mn and Si help tailor the SFE of the alloy which in turn determines the deformation-induced metastability of the material [12]. Lenhoff et al. reported that the addition of 2.5 wt% to an austenitic steel (11% Cr-15%Ni-1.1% Mn steel) decreased the SFE by 6–7 mJ/m^2^ [13]. Silicon addition to high Mn-steel resulted in a decrease of the SFE in the range of 1–3.5 mJ/m^2^ per wt% of Si [14]. Therefore, the addition of silicon helps tailor the SFE to achieve the transformation-induced plasticity. Furthermore, the addition of silicon also helps improve the electrochemical activity for passivation. A silicon-rich 18Cr-15Ni-3.5Si austenitic stainless steel spontaneously formed a protective passive film comprising mixed chromium and silicon oxides, which ensured better corrosion resistance in hot nitric acid [15]. The addition of silicon to duplex stainless steel was observed to improve pitting resistance by reducing the number of pits initiated at the grain boundaries due to SiO_2_-type film formation [16].

Due to the addition of Mn and Si, the Fe_42_Mn_28_Co_10_Cr_15_Si_5_ alloy exhibited a dual-phase microstructure comprising an *fcc γ*-phase and an *hcp ε*-phase with the thermodynamically predicted volume fractions of 63% and 37%, respectively [8,17]. Recently, Jin et al. reported a novel route of manufacturing CoFeNiMn HEA by a direct reduction of corresponding oxides. This study demonstrated that the direct reduction of MnO_2_ to metallic Mn was possible by offsetting the positive free energy of reduction with a negative enthalpy of mixing of Mn with other alloying elements, combined with high configurational entropy [18]. Depending on the processing conditions, the grain size and the volume fraction of the phases varied. The cast material showed a lower volume fraction of the *ε*-phase due to faster cooling [8]. Hot rolling of the cast-slab followed by annealing resulted in 52% *hcp* martensite and 48% *fcc* phase [17]. The friction stir processing of the Fe_42_Mn_28_Co_10_Cr_15_Si_5_ alloy resulted in a lower fraction of martensite (39%) and a higher amount of the *fcc* phase, accompanied by a significant reduction in grain size (from 120 μm to 5 μm) [17]. The strain hardening rate (d*σ*/d*ε*) of Fe_42_Mn_28_Co_10_Cr_15_Si_5_ having a grain size of 120 μm was reported as 6100 MPa, while the finer grain (5 μm) material showed a strain hardening rate of 8070 MPa. The strain hardening exponent, *n*, based on Hollomon’s relation: *σ = kε^n^*, was in the range of 0.33–0.44. The strain hardening behavior of the Fe_42_Mn_28_Co_10_Cr_15_Si_5_ is superior to that of Hadfield steel. The reported strain hardening rate of Hadfield steel was 5000 MPa at the early stages of the plastic deformation, which decreased to about 2000 MPa at a strain of about 0.06 and increased to about 3000 MPa at a strain of 0.24 [19]. Hutchinson and Ridley also reported similar strain hardening rates for a hot-rolled bar of Hadfield steel containing by weight 1.02%C, 13.9%Mn, 0.33%Si, 0.026%S and 0.052%P under tensile and compressive load conditions [20].

Machining is required for the manufacturing of components with tight dimensional tolerance in various engineering applications. Machining the highly work-hardenable materials using conventional processes is complex [21]. The poor machinability of strain-hardening materials is attributed to the high surface strains formed during the conventional metal cutting process, which results in the formation of defects such as twinning and stacking faults, leading to martensitic phase transformations. The strain hardening results in increased power requirement, enhanced tool wear, poor surface finish, diminished tool life and sometimes the breaking of the tools [22,23]. To overcome these limitations, unconventional machining processes such as electric discharge machining and electrochemical machining are adapted for machining the strain-hardening materials [24,25].

Electrochemical machining involves controlled removal of material by anodically dissolving the surface exposed to an electrolyte [26]. A DC potential is applied between the workpiece, which is connected to the positive terminal (anode), and a pre-shaped non-contact tool, acting as the cathode or counter electrode. The distance between the workpiece and the cathode is tightly controlled to achieve the required configuration of the machined surface. The machining rate (material removal rate) and surface finish are determined by the electrochemical parameters, including applied potential, current density, electrolyte, and temperature [27]. Four major material modifications were identified during the electrochemical machining process, namely, formation of flow grooves, passivation, pitting corrosion, and selective dissolution [28]. Among these, passivation affects the material removal rate while other modifications affect the quality of the surface finish [29].

FeCrNiCoMn high-entropy alloy and its equiatomic subsystems are considered promising candidates for structural materials in gas turbine engines [30]. The advanced gas turbine and aero-engine components require complex shapes meeting high standards of machining accuracy and surface finish. Electrochemical machining of gas turbine and aero-engine components has shown more advantages in processing quality, efficiency, and cost [31]. Klink et al. [24] reported electrochemical machining of three different FeMnNiCoCr types of HEAs in 20% sodium nitrate electrolyte at 15 V. Pitting or inhomogeneous dissolution was observed when the Co and Cr contents were low. Machining at a higher current density was recommended to minimize the pitting [24].

The selection of alloy composition Fe_42_Mn_28_Cr_15_Co_10_Si_5_ was based on the dual phase Fe_50_Mn_30_Co_10_Cr_10_ HEA proposed by [4]. The Cr content was increased at the expense of Fe and Mn because Cr increases the driving force for *γ → ε* transformation while Mn has the opposite effect. As reported by Nene et al., adding Si decreased the equilibrium fraction of *γ*, reaching a plateau at 5 at% Si, beyond which the effect was not significant [8]. The 5% Si addition was considered to result in the highest *γ → ε* transition temperature of 450 °C, and increased metastability of the *γ* phase by lowering the SFE. The Fe_42_Mn_28_Cr_15_Co_10_Si_5_ alloy exhibited higher yield strength than the Fe_50_Mn_30_Co_10_Cr_10_ HEA [4]. The corrosion resistance could also be higher for the Fe_42_Mn_28_Cr_15_Co_10_Si_5_ alloy due to silicon addition, higher Cr content, and lower Mn content compared to Fe_50_Mn_30_Co_10_Cr_10_ HEA. In this work, the electrochemical machining of the highly strain-hardenable Fe_42_Mn_28_Co_10_Cr_15_Si_5_ is investigated in non-chloride containing electrolytes to understand the influence of passivation and selective dissolution on the material removal rate and surface finish.

## 2. Materials and Methods

### 2.1. Material

HEA with a nominal composition of Fe_42_Co_10_Cr_15_Mn_28_Si_5_ (at%) was produced by vacuum arc-casting under an inert argon atmosphere at Sophisticated Alloys Inc., Butler, PA, USA. The cast slab was hot-rolled to a 20% thickness reduction, resulting in a plate of 13 mm thickness. 1 cm × 1 cm samples were band-sawed from the bar stock. One side of the sample was abraded with 120-grit sandpaper, cleaned, and soldered with a shielded copper wire for electrical connection. The sample with the soldered wire was mounted using epoxy resin, leaving only one side exposed with a surface area of 1 cm × 1 cm for further processing. The exposed surface was metallographically polished using a series of emery papers 120, 240, 400, 600, 800 and 1200 grit in that order. The polishing was performed in wet conditions by a continuous flow of water. The specimen was rotated by 90° when the polishing continued with the following higher grit emery paper. Several samples were prepared using this protocol.

### 2.2. Electrolytes

Both halide-type (NaCl) and non-halide-type (NaNO_3_) electrolytes were examined. Since high concentrations of anions lead to high-efficiency polishing, four types of electrolytes were tested: (1) 20 wt% NaNO_3_, (2) 20 wt% NaNO_3_ + 0.01 M HNO_3_, (3) 20 wt% NaNO_3_ + 0.01 M citric acid, and (4) 20 wt% NaCl. Lower ionic strength electrolytes were also studied to compare electrochemical behavior. These include (a) 1 M NaNO_3_, (b) 1 M NaNO_3_ + 0.01 M HNO_3_, and (c) 1 M NaNO_3_ + 0.01 M citric acid. The compositions of the electrolytes were selected based on the literature reports [32,33].

### 2.3. Electrochemical Characterization

To understand the passivation, etching, and dissolution behaviors of the HEA samples, electrochemical measurements were performed using a three-electrode configuration. The working electrode was the HEA sample. A Pt wire spiral was used as the counter electrode (surface area ~3 cm^2^). A silver chloride-coated silver (Ag/AgCl) wire immersed in the saturated potassium chloride solution was used as a reference electrode. A computer-controlled potentiostat (Gamry Interface 1000, Gamry Instruments Inc., Warminster, PA, USA) was used for electrochemical measurements. In a typical test, the open circuit potential (OCP) of the sample was recorded for 30 min, followed by electrochemical impedance spectroscopy (EIS). The EIS was performed at OCP by superimposing an AC signal of 10 mV and scanning the frequency from 10,000 Hz down to 0.01 Hz, with a scan rate of 5 points/decade. Subsequently, a potentiodynamic polarization test was conducted by scanning the potential from −0.5 V versus OCP (E_oc_) to 10 V versus E_ref_ at a scan rate of 2.5 mV/s. These polarization measurements were performed in 1 M NaNO_3_ solution at a scan rate of 0.5 mV/s, stopping at 1.6 V.

### 2.4. Electrochemical Machining

The electrochemical machining trials were performed using a two-electrode configuration and a potentiostat (Gamry Interface 1000, Gamry Instruments Inc., Warminster, PA, USA). In this study, the epoxy-mounted HEA sample was used as a working electrode (anode). The counter electrode (cathode tool) was an insulated Pt wire. A 0.5 mm diameter Pt wire was masked using epoxy, exposing only the end circular section. In some experiments, a Pt wire with a diameter of 120 μm was used. This insulated wire was connected to the counter electrode and reference electrode terminals of the potentiostat. The HEA sample and Pt electrode were immersed in the test solution, and the distance between the HEA surface and Pt end was controlled at 1 mm using a micrometer-controlled Z linear stage (Make: Keenso, China Model: SEMZL60-ACR). Dissolution tests were performed under potentiostatic conditions at 5 V, 7.5 V, and 10 V for a total duration of 1 h. Every 15 min, the test was interrupted, and the samples were removed from the solution, washed, dried, and examined under a confocal microscope to measure the dimensions (diameter and depth of the hole formed) of the material removed. After the microscopic analysis, the potentiostatic test was resumed in the same solution for one hour. All tests were duplicated or triplicated to ensure reproducibility and enable statistical analysis. Each test started with a freshly polished sample, and fresh electrolyte.

### 2.5. Characterization of Tested Samples

The microstructures and surface morphologies of the samples before and after electrochemical tests were analyzed using a confocal Raman microscope (HORIBA XploRA Plus, Horiba Ltd., Kyoto, Japan), equipped with a 532 nm, 100 mW, Class 3B internal laser and a CCD detector (Model: Syncerity). X-ray diffraction (XRD) analysis was conducted using a Rigaku SmartLab diffractometer (Rigaku Holdings Corporation, Tokyo, Japan) with a Cu-Kα radiation source (λ = 1.5406 Å), operated at 40 kV and 44 mA. Diffraction patterns were collected in the 2θ range of 20° to 90°, with a step size of 0.01° and a scan rate of 2°/min. The crystalline phases identified from the XRD patterns were matched with standard data from the ICDD PDF-2 database (Release 2016). The *ε*-martensite phase corresponds to the Iron–Manganese (Fe_0.8_Mn_0.2_) phase with JCPDS card no. 01-071-8285, having a hexagonal close-packed structure (space group: P6_3_/mmc). The austenite (*γ*) phase corresponds to γ-Fe, with JCPDS card no. 01-071-4649, exhibiting a face-centered cubic (space group: Fm-3m) structure. XPS analysis was performed at a commercial laboratory (InnovaTECH, Plymouth, MN, USA). The XPS data were collected using a monochromatic Al Kα X-ray source at a take-off angle of 65° and an analyzed area approximately 1 mm in diameter. Low-energy resolution survey scans were obtained from each sample to identify the elements present. The atomic concentrations of these elements and their local chemistries were determined from higher energy resolution multiplex scans. The step size for the high-resolution scans was 0.2 eV. The high-resolution data were smoothed by the 7-point Savitsky-Golay smoothing function. Charge correction was carried out by considering the C *1s* peak at 284.8 eV.

## 3. Results and Discussion

### 3.1. Microstructure

**Figure 1:**
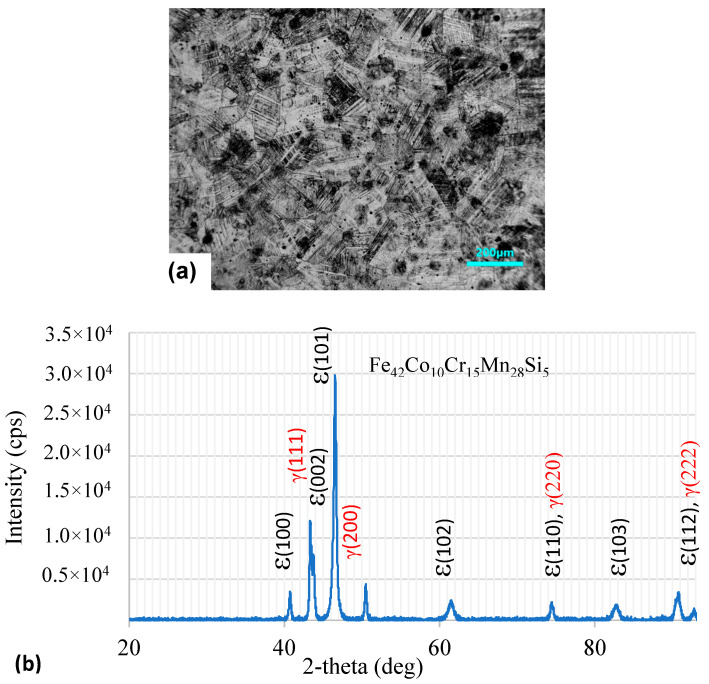
(**a**) Optical microstructure of Fe_42_Mn_28_Co_10_Cr_15_Si_5_ in as-received condition. The hcp-ε phase distribution in the *fcc*-*γ* matrix is revealed due to the hot-rolled condition. (**b**) XRD pattern of the Fe_42_Co_10_Cr_15_Mn_28_Si_5_ in the hot-rolled condition. Peaks corresponding to *ε*-martensite and austenite (*γ*) phases are observed.

Figure 1a shows the optical microstructure of the Fe_42_Co_10_Cr_15_Mn_28_Si_5_ sample (hereafter referred to as HEA sample). The microstructure revealed the presence of both the *fcc γ*-phase (lightly etched) and *hcp-ε* phase (dark etched). The grain size was in the range of 100–140 μm. The dual-phase microstructure was confirmed by the XRD analysis, as shown in Figure 1b. The XRD peaks observed at 2θ values 43.5, 50.7, 74.5, and 90.8° could be associated with the (111), (200), (220), and (222) crystal planes of austenite (*γ*-phase), respectively [34]. The diffraction peaks at 40.7, 43.8, 46.5, 61.6, 74.5, 82.9, and 90.8 could be assigned to the (*hkil*) planes (10-10), (0002), (10-11), (10-12), (11-20), (10-13), and (11-22) of the *ε*-martensite phase [35]. There were a couple of overlapping peaks associated with both phases. Significant peak broadening was observed, which could be attributed to the smaller crystallite size of the transformed *ε*-martensite in the austenite matrix. Using Scherrer’s equation (t = 0.9λ/FWHM. cos θ) [36], the crystallite size of the *ε*-phase was estimated to be around 0.23 μm. The ratio of the phases present in the alloy was calculated by two methods. In method 1, the intensities of all the peaks were numerically integrated and the fraction of the *ε*-phase was calculated using the relation:

(1) $$Fraction\;of\;\varepsilon \;phase=\;\frac{\sum_{\varepsilon }I_{\left(hkl\right)}}{\sum_{\gamma }I_{\left(hkl\right)}+\sum_{\varepsilon }I_{\left(hkl\right)}}$$

where *I* = integrated intensity of the XRD peaks. Method 2 for estimating the volume fraction of phases is discussed in the Supplementary Materials. The estimated volume fraction of the *ε*-phase based on the integrated intensity ratios of both phases was about 0.79. The higher volume fraction of the *ε*-martensite was due to the hot-rolled condition of the material in the as-received condition. The dark etching of the *ε*-phase indicated that the HNO_3_:HCl (3:1 by volume) etchant preferentially attacked the martensitic regions.

### 3.2. OCP and Impedance

**Figure 2:**
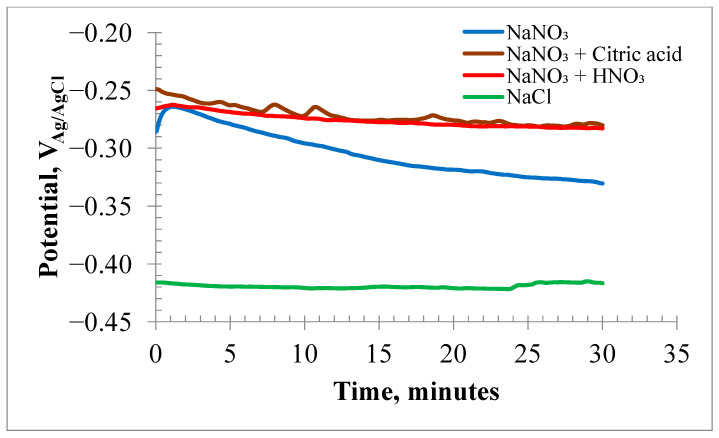
Open circuit corrosion potential plots for HEA samples submerged in electrolytes of 20% NaNO_3_, 20% NaNO_3_ + 10 mM Citric acid, 20% NaNO_3_ + 10 mM HNO_3_ and 20% NaCl.

Figure 2 displays the OCP values of the HEA samples in various high-concentration electrolytes. The OCP in the neutral NaNO_3_ solution initially shifted to less negative values. After a few minutes, the potential shifted to more negative values, ranging from −262 mV to −330 mV. Conversely, the OCP values in the acidified nitrate solutions changed from −260 mV to −282 mV within 30 min. The 20% NaCl solution showed a more active corrosion potential of −420 mV.

**Figure 3:**
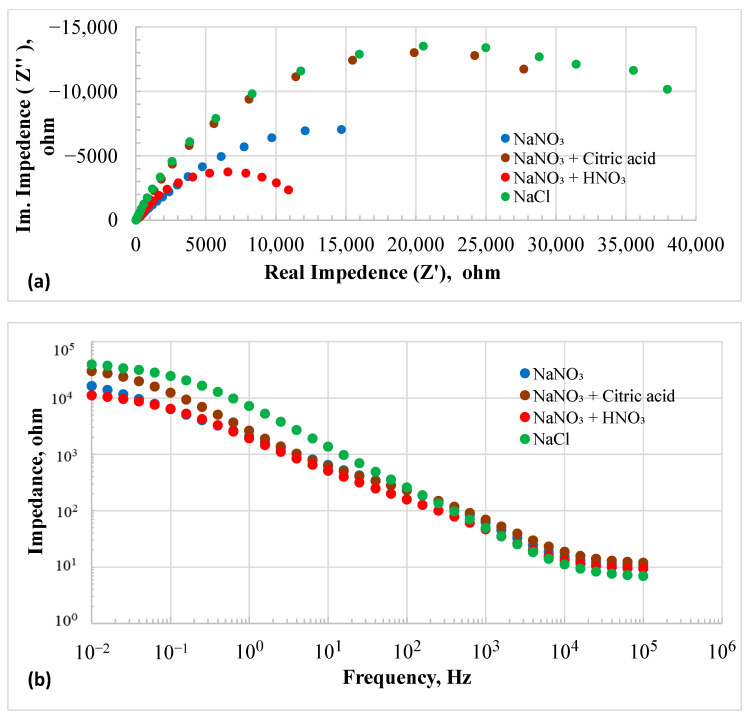
Electrochemical Impedance spectroscopy plots of HEA in different electrolytes at OCP: (**a**) Nyquist plots and (**b**) Bode plots.

The OCP of the HEA in the NaCl solution remained nearly constant at −420 mV over time. This more negative OCP suggested that the surface film on the HEA was less stable in chloride than in nitrate solutions. Figure 3a,b display the EIS results at OCP as Nyquist and Bode plots, respectively. EIS data modeled with EEC are discussed in the Supplementary Materials.

The EIS results of all the conditions were fitted with a three-time constant electrical equivalent circuit (EEC) as shown in Figure S1, and model parameters are listed in Table S2. In this EEC diagram, *R*_0_ represents electrolyte resistance, which was the highest in the 20% NaNO_3_ + 0.01M citric acid solution and the lowest in the 20% NaCl solution. The EEC consists of three capacitive loops and one inductive loop. The inductive loop represents the adsorption of the anions on the surface of the electrode [37]. The impedance of an inductor is given as *Z = j*2*πfL*, while the impedance of a capacitor is *1*/*(j2πfC)*. Therefore, as the frequency approaches zero, the impedance of the inductor is negligible, and the impedance of the capacitor reaches infinity. At low frequencies (reaching the DC condition), the resistance can be simplified as [37]:

(2) $$\frac{1}{R_{p}}=\frac{1}{R_{1}+R_{2}+R_{4}}+\frac{1}{R_{3l}}$$

The *Q*_1_*R*_1_ loop can be associated with the overall electrochemical interface structure comprising the Electrode/electrolyte macroscopic electric double layer. This outer loop contains an inner loop of *Q*_2_*R*_2_, which could represent the double layer due to roughness of the surface. This inner loop contains another loop of *Q*_4_*R*_4_, which could be associated with the microscopic pores within the surface asperities. It is noted that the inductance is the lowest in the 20% NaCl solution and highest in the 20% NaNO_3_+ 0.01 M HNO_3_ solution. The addition of citric acid increased the inductance as compared to the 20% NaNO_3_ without any addition. Overall, the results suggest that nitrate solutions promote adsorption on the electrode surface. The 20% chloride solution showed higher values of *R* and *Q* in all the loops than those of the other solutions. The slope of the Bode plot between frequencies 10^4^ and 1 Hz was 0.7, indicating that the leaky capacitor of the RC circuit could be expressed as *C_L_* = *I. t^n^*/*V*, where *n* equals 0.7. Here, *C_L_* represents the capacitance of the leaky capacitor, *I* is the current, *V* is the voltage, and *t* is time. If *n* equals 1, it signifies an ideal capacitor. When *n* = 0.5, conduction is diffusion-controlled, and the capacitor-like behavior resembles a Warburg element [38]. The impedance in the chloride solution was the highest among all the tested solutions. The addition of citric acid resulted in high impedance values, whereas the lowest impedance was observed in the nitrate solution with added nitric acid.

### 3.3. Electrochemical Polarization Behaviors

**Figure 4:**
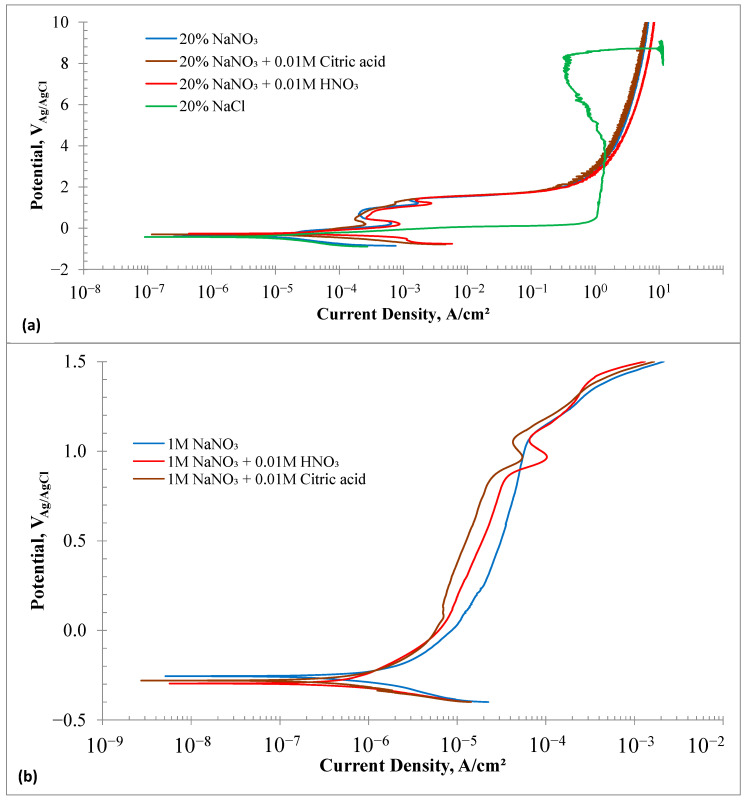
(**a**) Potentiodynamic Polarization plots of HEA samples in electrolytes of 20% NaNO_3_, 20% NaNO_3_ + 10 mM Citric acid, 20% NaNO_3_ + 10 mM HNO_3_ and 20% NaCl solutions at room temperature. (**b**) Potentiodynamic Polarization plots of HEA samples in electrolytes of 1 M NaNO_3_, 1 M NaNO_3_ + 10 mM Citric acid, and 1 M NaNO_3_ + 10 mM HNO_3_ solutions at room temperature.

Figure 4a shows the potentiodynamic polarization measurement results of the HEA samples in different electrolytes. The overall polarization behavior can be categorized into two distinct groups: (i) formation of a passive layer, and (ii) no passivation. The polarization behavior in the chloride solution was different from that observed in the nitrate-based solutions. Even though the impedance was high in the chloride solution at OCP, the anodic current increased monotonically with the applied potential. The current reached a value of about 1 A/cm^2^ at 0.5 V. Increasing the potential did not result in a sharp increase in the current until about 4 V, where the maximum anodic current density was about 1.45 A/cm^2^. Further increase in the potential resulted in a decrease in the current density, possibly due to localized supersaturation of the dissolved species and reprecipitation of a salt layer, which limited the current density. The current started to increase sharply again beyond 8.2 V. The experiment was interrupted at 9 V as the current was beyond the capacity of the potentiostat. Essentially, no passivation behavior was observed in the 20% chloride solution.

The polarization behavior of HEA samples in nitrate-based solutions was similar. Two dissolution peaks appeared at 0.18 V and 1.2 V. Adding nitric acid resulted in the highest first dissolution peak current density of 0.8 mA/cm^2^ and a second peak current density of 2.75 mA/cm^2^. It was expected that adding citric acid as a chelating agent would boost the dissolution current density. However, the citric acid + nitrate solution showed the lowest dissolution peak current density among the three nitrate solutions. The initial dissolution peak at 0.2 V likely corresponds to the oxidation of elements (Fe, Mn, Co, and Cr) to their divalent states. Between 0.4 V and 0.9 V, a passivation-like behavior was observed, where oxidized metal ions formed a surface layer and reduced the current density to about 0.25 mA/cm^2^. The second dissolution peak may be linked to the oxidative dissolution of metal cations in the surface film. All nitrate solutions showed a sharp rise in anodic current density at potentials above 1.4 V and up to 1.9 V, with similar current densities. The increase in current density was less notable beyond 2 V. At 2 V, the current density was 0.3 A/cm^2^, rising to 8.4 A/cm^2^ at 10 V. Among the nitrate solutions, nitric acid addition produced the highest current density.

Figure 4b displays the potentiodynamic polarization curves of the HEA samples tested in low-concentration (1 M vs. 2.35 M) nitrate solutions. The *E_corr_* values of the HEA samples in 1 M NaNO_3_ solutions were slightly more positive than those in 20% nitrate solutions. Only one dissolution peak appeared in the acidified 1 M NaNO_3_ solutions, occurring at approximately 0.96 V. The dissolution peak current was an order of magnitude lower in the 1 M NaNO_3_ solution compared to the 20% nitrate solution. The HEA samples exhibited clear passivation behavior in 1 M nitrate solutions within a potential range of 0.1–0.9 V. The addition of citric acid resulted in the lowest average passive current density of 15 μA/cm^2^, while nitric acid showed the highest average passive current density of 35 μA/cm^2^. The passivation current densities were an order of magnitude lower than those in the 20% nitrate solutions. The passivation behavior observed in the 1 M NaNO_3_ solutions suggests that lower concentrations of NaNO_3_ are not suitable for electrochemical machining of HEA components.

Table 1 presents a summary of the electrochemical parameters obtained from the polarization measurements. It includes various parameters such as corrosion potential (*E_corr_*), corrosion current density (*i_corr_*), exchange current density for the cathodic reaction (*i_oc_*), exchange current density for the anodic reaction (*i_oa_*), and Tafel slopes for both cathodic and anodic polarization. The exchange current density for the cathodic reaction was calculated specifically for the oxygen reduction reaction. The redox potential of oxygen reduction depends on pH for the reactions listed below.

Acidic: pH: 4H^+^ + O_2_ + 4e^−^ → 2H_2_O; E = 1.23 − 0.059 pH V_SHE_ (3)

Neutral: 2H_2_O + O_2_ + 4e^−^ → 4OH^−^; E = 0.396 − 0.059 log [pH-14] V_SHE_ (4)

Table 2 shows the initial pH of the test solutions and the corresponding redox potentials for the oxygen reduction reaction (ORR). The exchange current density for the ORR was calculated by extrapolating the linear part of the cathodic polarization plot until it intersected the redox potential of the ORR. The oxidation of iron was considered the main anodic reaction. The exchange current density was determined by evaluating the redox potential of the reaction.

Fe → Fe^2+^ + 2e^−^; E = −0.44 + 0.0295 log (Fe^2+^)  V_SHE_ (5)

It is observed that the exchange current densities strongly depend on the Tafel slopes. The steeper the Tafel slope, the higher the exchange current densities. Electrolytes with low concentrations showed smaller cathodic exchange current densities and smaller Tafel slopes. Except for the nitric acid added to the 20% NaNO_3_ solution, other high-concentration electrolytes exhibited nearly identical exchange current densities. The corrosion current densities of the high-concentration solutions were similar to each other, while those of 1 M NaNO_3_ solutions were an order of magnitude lower. The summarized electrochemical parameters will aid in predicting dissolution rates and optimizing process parameters through modeling dissolution kinetics.

### 3.4. Electrochemical Machining

**Figure 5:**
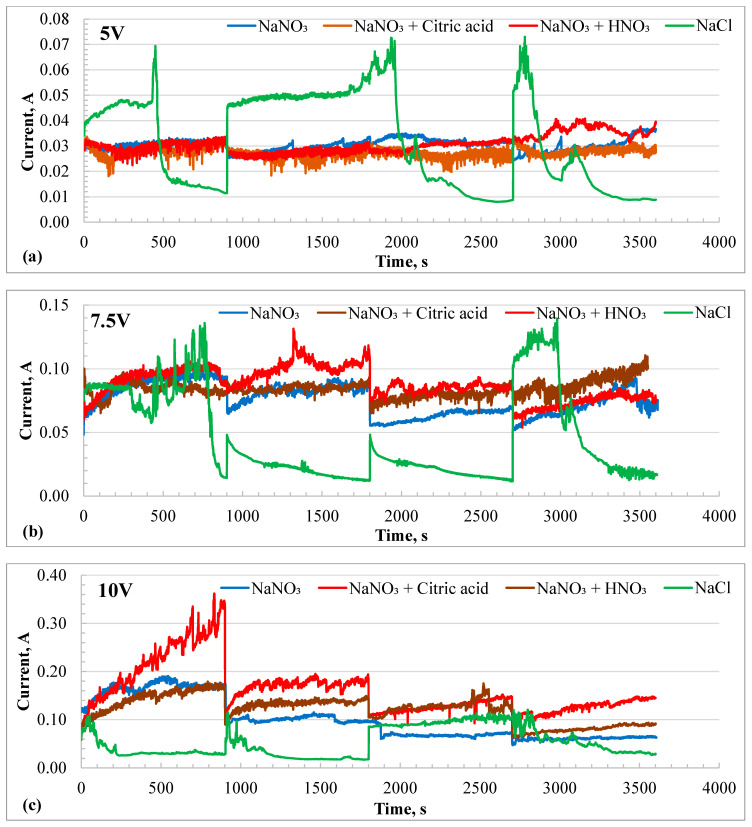
Current–time (*I*-*t*) profiles of HEA during potentiostatic electrochemical machining in different electrolytes at different potentials. (**a**) at 5 V, (**b**) 7.5 V, and (**c**) 10 V. The experiments were interrupted every 15 min to measure the depth of material machined. The current spikes are due to the interruptions.

The electrochemical machining of the HEA samples was performed at three different potentials, 5, 7.5, and 10 V, in each 20% nitrate-based electrolyte for a total duration of 1 h. After every 15 min, the experiment was interrupted to measure the volume loss by the dissolution process. The potentiostatic experiment restarted and continued for another 15 min. Figure 5a–c show the *I-t* profiles during the electrochemical machining (ECM) process at 5, 7.5, and 10 V, respectively. ECM at 5 V in nitrate-based solutions produced a current between 20 and 30 mA, while in the chloride solution, the current varied widely from 10 mA to 70 mA. Notable current spikes were observed in the chloride solution at all potentials when the ECM was restarted after each 15 min interval. The current transients exhibited a sharp increase at the start of the experiment, followed by a continuous decay, likely due to the formation of a surface salt layer.

**Figure 6:**
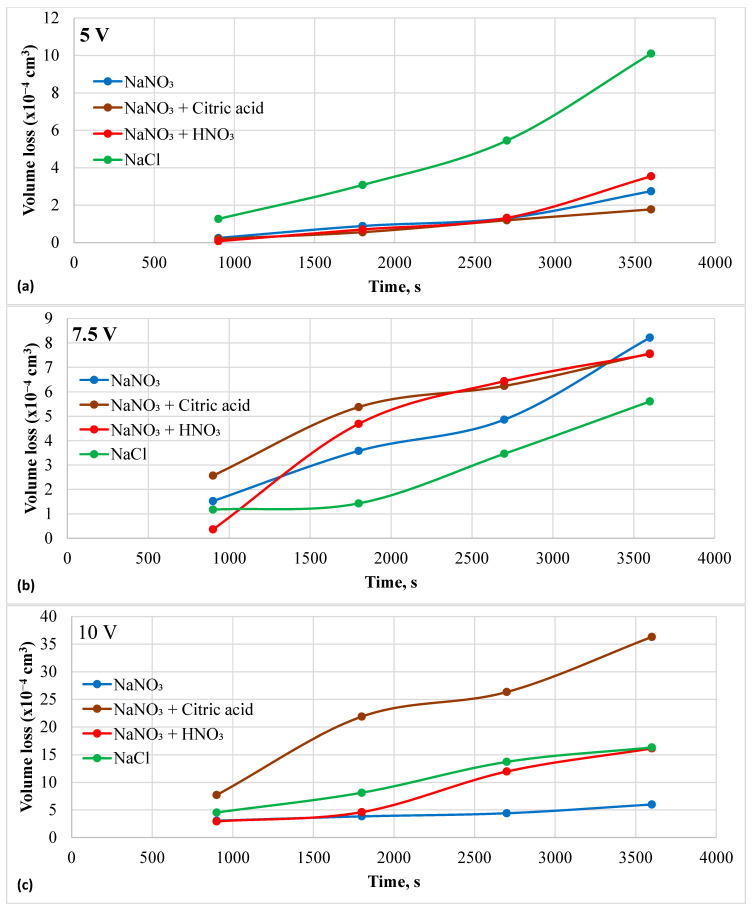
Material loss during electrochemical machining in different electrolytes and at different potentials. (**a**) at 5 V, (**b**) 7.5 V, and (**c**) 10 V.

The sample rested flat at the bottom of the beaker, with the insulated Pt wire counter electrode positioned just above it. The dissolved species were transported by natural convection caused by the electric field and heat generated through Joule heating. As a result, the supersaturated solution near the sample surface led to a decrease in current. The current spikes were less at 10 V in the chloride solution but were more prominent in the nitrate + citric acid solution. However, the current increased instead of decaying in the nitrate solutions. The current increased with the applied potential for all electrolytes, except in the chloride solution at 10 V. Here, only current, not current density, was reported because the affected area varied depending on the electric field distribution. The lower current in the chloride solution at 10 V could be attributed to a more focused electric field. To better understand the *I-t* profiles, the resulting volume loss due to charge accumulation was plotted over time and presented in Figure 6a–c. The highest volume loss during ECM at 5 V was recorded in the chloride solution. All nitrate solutions showed similar volume losses at 5 V. However, ECM at 7.5 V showed a reverse trend compared to that observed at 5 V. The chloride solution resulted in the lowest volume loss, while the neutral nitrate solution exhibited the highest volume loss after 1 h, with volume loss increasing linearly over time. At 10 V, the ECM in the nitrate + citric acid solution had the highest removal rate of material, followed by the chloride solution.

Table 3 summarizes the results of the potentiostatic ECM experiments. The total charge accumulated during one hour of ECM increased with applied potential. Theoretical volume loss related to the accumulated charge was calculated using Faraday’s law. The atomic weight of the HEA is 53.93 g/mol. The theoretical charge needed to dissolve one mole of Fe_48_Mn_28_Co_10_Cr_15_Si_5_ is 243,770.6 coulombs. The theoretical density is 7.7 g/cm^3^, and the molar volume is 7 cm^3^. Using this data, the theoretical volume loss was calculated based on the experimentally measured charge and is listed in the table. Faradaic efficiency was determined by dividing the experimental volume loss by theoretical volume loss. The nitrate-based solutions generally exhibited a lower Faradaic efficiency than the chloride solution. The Faradaic efficiency increased with the applied potential in both the citric acid + nitrate solution and the chloride solution. The other two electrolytes did not show a specific trend. The highest Faradaic efficiency observed in the nitrate-based solution was 21.9%, using citric acid at 10 V. This efficiency was reached at 7.5 V in the chloride solution. Overall, the chloride solution demonstrated higher Faradaic efficiency than the nitrate solution. The lower efficiency of the nitrate solution could be due to charge loss in other anodic reactions, such as oxygen evolution, and the formation of an oxide layer.

### 3.5. Morphologies After Dissolution

**Figure 7:**
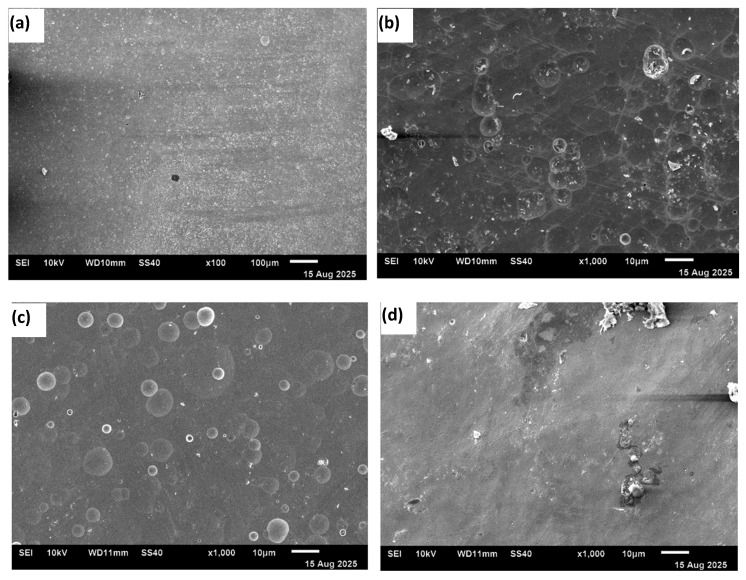
SEM images of the surface morphology of samples after potentiodynamic polarization scans in different electrolytes: (**a**) 20% NaNO_3_, (**b**) 20% NaNO_3_ + 10 mM HNO_3_, (**c**) 20% NaNO_3_ + 10 mM citric acid, and (**d**) 20% NaCl.

Figure 7a–d show scanning electron micrographs of the HEA sample surfaces after potentiodynamic polarization tests (final voltage ~10 V). No selective attack on specific phases or grain boundaries was observed in any of the electrolytes.

The material removal was generally uniform across the surface. However, a mirror-like surface free of debris was not achieved after the experiments. Gas bubbles adhered to the surface, causing uneven dissolution in the acidified nitrate solutions. Surface waviness was visible on the sample tested in the chloride solution. At higher potentials in 20% NaCl solution, the anodic behavior transitioned from activation-controlled to mass-transfer-controlled because, under static conditions, the dissolved ions reached supersaturation and reprecipitated on the electrode surface. The local pH changes also contributed to the precipitation of a salt layer. Additionally, oxygen evolution occurred at higher potentials. The oxygen bubbles blocked the surface, leading to a nonuniform surface condition. All these effects resulted in a reduction in current during the polarization. The surface waviness observed in Figure 7d serves as a morphological signature of these coupled processes—non-uniform dissolution, product accumulation, and local blocking—explaining the observed decrease in current.

**Figure 8:**
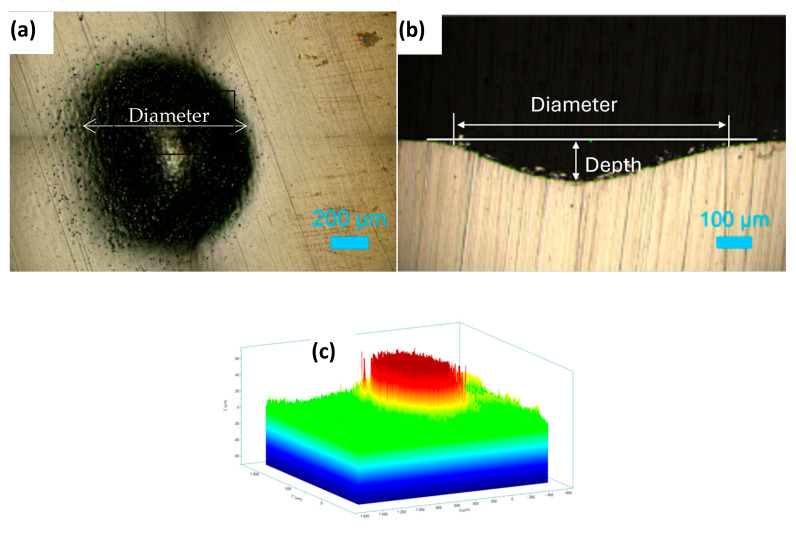
Electrochemical drilling of a hole in HEA by potentiostatic dissolution at 11 V in 1 M NaNO_3_ for 20 min (recorded current: 10–15 mA; counter electrode: Pt wire, 120 μm diameter). (**a**) Top-view image of the drilled hole (diameter ≈ 850 μm), (**b**) cross-sectional view showing hole depth ≈ 100 μm, and (**c**) corresponding depth profile.

**Figure 9:**
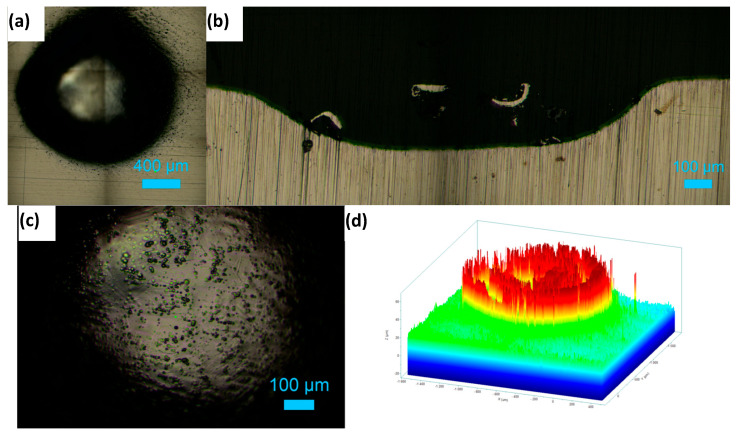
Electrochemical drilling of a hole in HEA by potentiostatic dissolution at 11 V in 1 M NaNO_3_ + 10 mM citric acid for 20 min (recorded current: 10–12 mA; counter electrode: Pt wire, 120 μm diameter). (**a**) Top-view image of the drilled hole (diameter ≈ 1587 μm), (**b**) cross-sectional view showing hole depth ≈ 234 μm, (**c**) image of the bottom surface, and (**d**) depth profile of the hole.

**Figure 10:**
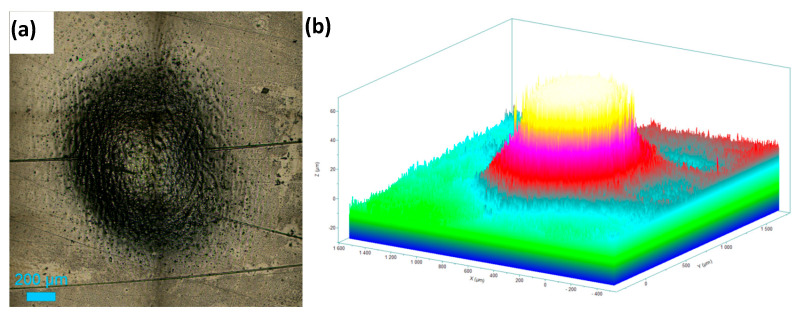
Electrochemical drilling of a hole in HEA by potentiostatic dissolution at 5 V in 1 M NaNO_3_ + 10 mM citric acid for 20 min (counter electrode: Pt wire, 120 μm diameter). (**a**) Top-view image of the drilled hole (diameter ≈ 503 μm), and (**b**) depth profile showing hole depth ≈ 70 μm.

Figure 8a shows the top surface of the hole machined in 1 M NaNO_3_ solution by applying a potential of 11 V for 20 min. The roughness variations at the bottom of the machined hole are presented in Figure 8b as a cross-sectional view of the hole. An inverted depth profile is shown in Figure 8c. Figure 9a displays the hole formed on the HEA surface at 11 V in the 1 M NaNO_3_ + 0.01 M citric acid solution. The cross-section of the hole is shown in Figure 9b. The bottom surface of the pit is shown in Figure 9c. The inverted depth profile of the hole is displayed in Figure 9d. The bottom surface lacked a good finish due to surface waviness and bubbles stuck to it, which affected uniform dissolution. Figure 10a shows the image of a hole machined in citric acid containing nitrate solution at 5 V. The inverted depth profile is shown in Figure 10b. ECM at a lower voltage resulted in a relatively curved rim of the hole.

No difference in the dissolution rate between the *fcc γ*-phase and *hcp ε*-phase was observed in this study during the potentiostatic test conditions at 5–10 V. The dissolution was found to be uniform. The reason for such uniform dissolution at high polarization conditions could be attributed to the similar chemical potentials of the *ε* and *γ* phases. The *γ → ε* phase transformation is a martensitic process governed by the Gibbs free energy change (ΔG_γ-ε_) of the metastable *γ* phase transforming into the martensitic *ε* phase. The ΔGγ-ε for the investigated Fe_42_Mn_28_Cr_15_Co_10_Si_5_ alloy is considered low due to the high metastability of the *γ* phase and low stacking fault energy [8]. This low ΔG_γ-ε_ results in a minor difference in chemical potential, so no significant galvanic coupling between the two phases is expected. Additionally, the martensitic transformation from *γ* to *ε* is not diffusion-controlled, resulting in no compositional difference between these phases. Shen et al. observed uniform elemental distribution across the fusion zone and the heat-affected zone of the fusion-welded Fe_42_Mn_28_Cr_15_Co_10_Si_5_ alloy [39]. The homogeneous elemental distribution without a distinct chemical gradient across phase boundaries is a characteristic of metastable HEAs. Therefore, no preferential dissolution of phases was observed during the ECM.

### 3.6. XPS Analysis

The surface of the HEA samples was analyzed by XPS to understand the chemical state of the elements present on the surface. The valence states of the constituent elements will be determined based on the binding energy of the 2*p* electrons. The *2p*_3/2_ spectrum of the ionized first transition elements such as Cr, Mn, Co, and Fe can be split into multiple peaks. When the paramagnetic ions (with unpaired electron configuration) are photoionized, they are excited to a few eV above the ground state, which reduces the kinetic energy of the emitted photoelectron. This results in the appearance of a shake-up or satellite peak at a higher binding energy than the main peak [40]. Multiple splitting occurs when an ion has unpaired electrons. For example, unpaired electrons are available in Cr(III) with an electron configuration *[Ar] 3d^3^*, and in Fe(III) with a configuration of *[Ar]3d^5^*. When photoionization during XPS analysis results in a core electron vacancy, the unpaired core electron couples with unpaired outer shell electrons of the transition metal ions, leading to multiple splitting [41]. Satellite peaks were absent in most of the spectra. The mixed valent Fe_3_O_4_ will not show a satellite peak. If Fe^2+^ is present as FeO, then a satellite peak at 6 eV above the *2p*_3/2_ line will be present [42]. A satellite peak at 8 eV above the *2p*_3/2_ will be observed when Fe^3+^ is present as Fe_2_O_3_ [43]. Similarly, Mn^2+^ will reveal a satellite peak at 5.4 eV from the main peak [44]. Presence of Co_3_O_4_ will be marked by a weak satellite peak appearing above 10 eV from the main peak [45]. The absence of satellite peaks in this study indicated that the oxides present on the samples were not pure FeO, Fe_2_O_3_, MnO, or Co_3_O_4_. These results are in alignment with the previous studies on the passivation of Fe_42_Mn_28_Cr_15_Co_10_Si_5_ alloy in 3.5% NaCl solution [35,46].

The Cr(III), present in Cr_2_O_3_, will have five multiple peaks of equal FWHM (0.9 eV) in the range of 575.7–578.9 eV [47]. The Cr(0) is characterized by one asymmetric peak with a FWHM of 0.9 eV in the range from 573.9 to 574.5 eV. The *2p*_3/2_ peak of Fe(0) occurs at 706.6 eV with a FWHM of 0.88 eV, and the 2 *p*_1/2_ peak is at 719.7 eV. The *2p*_3/2_ spectrum of Fe(III) in Fe_2_O_3_ could split into six peaks occurring at 709.8, 710.8, 711.6, 712.7, 713.7, and 719.3 eV [42]. The Mn(0) exhibits a 2*p*_3/2_ peak at 638.64 eV with a 2*p*_3/2_ to 2*p*_1/2_ splitting of 11.10 eV [48]. The 2*p*_3/2_ spectrum of Mn(II) in MnO has six peaks in the range of 640.2–645.9 eV, while the 2*p*_3/2_ spectrum of Mn(III) in Mn_2_O_3_ has five peaks in the range of 640.8–646.2 eV [47]. The 2*p*_3/2_ spectrum of Co(0) shows three peaks in the range of 778.1–783.1 eV, while the Co(II) in CoO shows four peaks in the range of 780–786.5 eV. The 2*p*_3/2_ spectrum of Co(III) in CO_3_O_4_ shows four peaks in the range of 779.6–785.2 eV [47].

**Figure 11:**
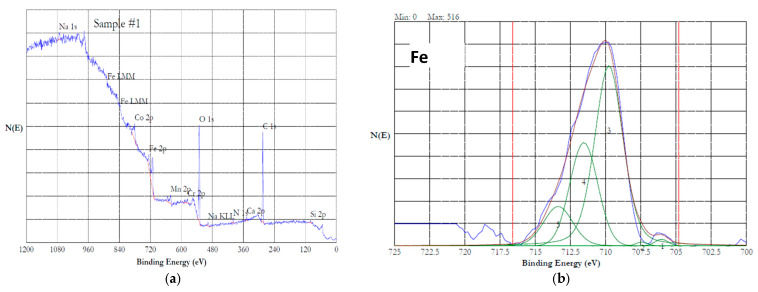

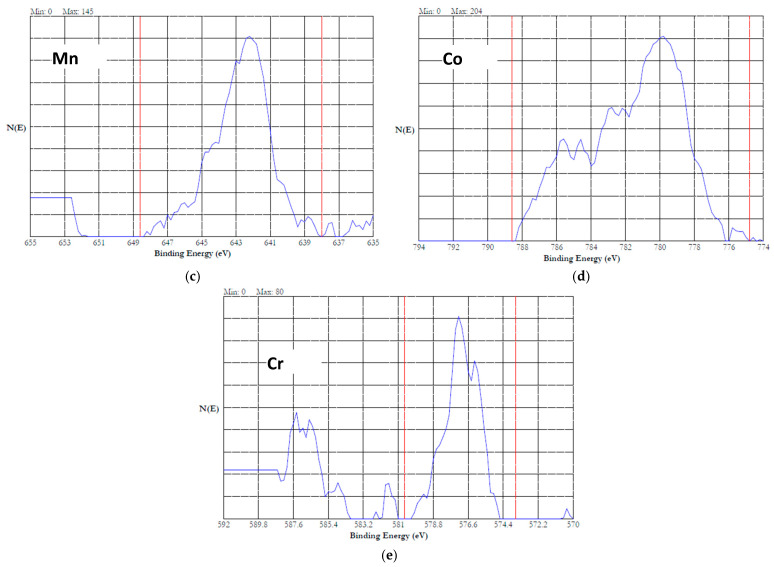
XPS results of HEA surface passivated at 1 V in 20% NaNO_3_ solution for 1 h. (**a**) survey spectrum, (**b**–**e**) high resolution spectra of iron (**b**) (the peak is split into five peaks and numbered 1 to 5), manganese (**c**), cobalt (**d**) and chromium (**e**).

The XPS data of this investigation were analyzed using the above literature values. Figure 11a shows the survey spectrum of the XPS of HEA passivated in 20% NaNO_3_ at 1 V for one hour. The spectrum indicates the presence of all the alloying additions. Figure 11b–e show the high-resolution 2*p*_3/2_ spectra of Fe, Mn, Co and Cr, respectively. The 2*p*_3/2_ spectrum of Fe (Figure 11b) could be split into five peaks, indicating that iron was in the oxidized state of Fe(III). A satellite peak at 718 eV, which was 8 eV above the 2*p*_3/2_ peak at 710 eV, suggested that the iron was present as Fe_2_O_3_ in the passivated HEA surface [43]. The 2*p*_3/2_ spectrum of Mn is displayed in Figure 11c. This spectrum could be resolved into five peaks, indicating that the Mn was present in the oxidized state. Absence of a distinct satellite peak at ~5.4 eV above the binding energy of the main peak suggested that the Mn was in the form of Mn(III) [44]. Figure 11d shows the 2*p*_3/2_ spectrum of cobalt, which can be resolved into multiple peaks. The peaks at 780, 782, 785.6, and 786.5 suggest that the Co was present as Co(II) [47]. The 2*p*_3/2_ spectrum of Cr is presented in Figure 11e. This spectrum can be resolved into five peaks, which indicates that the Cr was present as Cr(III). The presence of Cr(OH)_3_ can be ruled out because Cr(OH)_3_ will have a single peak at 577.3 eV. The peaks resolved at 575.4, 576.4, 577.5, 578.6, and 579 could be attributed to the Cr(III) in the form of Cr_2_O_3_ [47]. The presence of FeCr_2_O_4_ is ruled out due to the presence of five peaks instead of four. Overall, potentiostatic passivation at 1 V in the neutral nitrate solution resulted in 100% oxidation of the constitutive elements.

**Figure 12:**
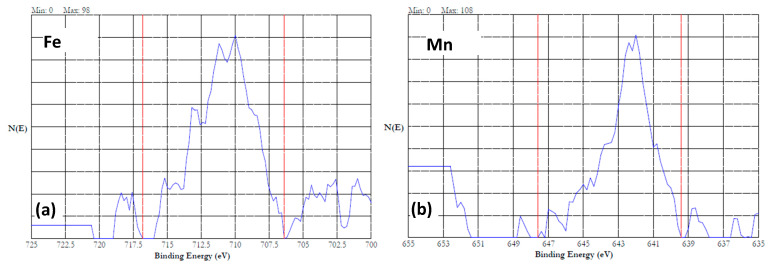
High resolution XPS results of HEA surface passivated at 0.7 V in 20% NaNO_3_ + 0.01 M citric acid solution for 1 h. (**a**) iron and (**b**) manganese.

**Figure 13:**
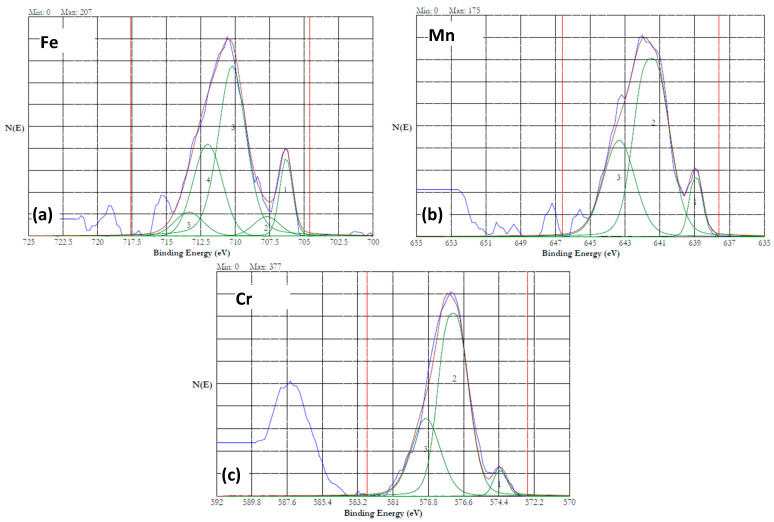
High resolution XPS results of HEA surface dissolved at 5 V in 20% NaNO_3_ solution for 15 min. (**a**) iron (**b**) manganese, and (**c**) chromium. No cobalt signals were observed. The peaks are resolved into multiple peaks, and the multiplets are numbered.

Figure 12a,b show the 2*p*_3/2_ spectra of the Fe and Mn of the HEA passivated at 0.7 V in the 20% NaNO_3_ + 0.01 M citric acid solution. Interestingly, cobalt and chromium were not detected on the surface. The Fe and Mn were in fully oxidized states as discussed earlier. Figure 13a–c show the high-resolution XPS data of the HEA sample conditioned at 5 V in the neutral nitrate solution. Cobalt was not detected in this sample. The iron, manganese, and chromium were in the form of both metal and oxidized ions. Figure 12a shows the iron 2*p*_3/2_ spectrum, which contains Fe(0) due to a peak at 706.4 eV and Fe(II) and Fe(III) due to the peak at 711.4 eV [46]. Figure 12b shows the Mn 2*p*_3/2_ spectrum, which contains a peak at 638.8 eV corresponding to Mn(0), and a peak at 641.8 eV associated with Mn(III) [46]. The Cr 2*p*_3/2_ spectrum is displayed in Figure 13c, which has a minor peak at 574.2 eV associated with Cr(0). A prominent peak at 577.2 eV and the 2*p*_1/2_ peak at 587.2 eV indicate the presence of Cr(III) as Cr(OH)_3_ [49].

**Figure 14:**
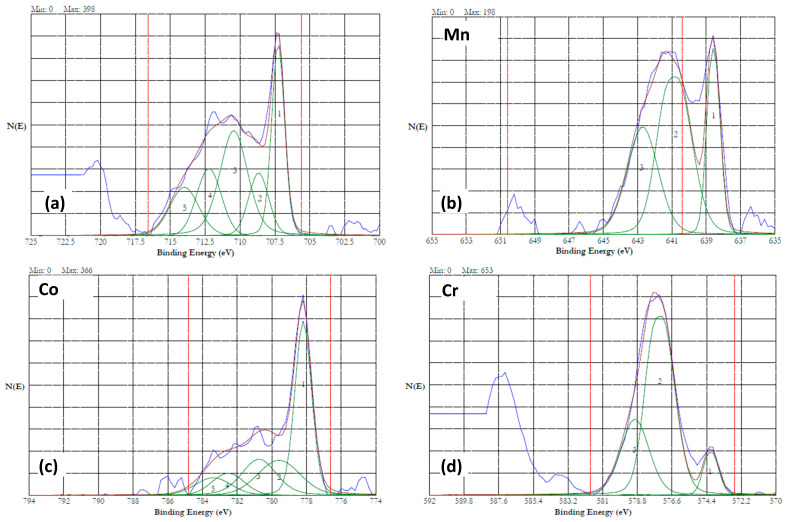
High resolution XPS results of HEA surface dissolved at 5 V in 20% NaNO_3_ + 0.01 M citric acid solution for 15 min. (**a**) iron (**b**) manganese, (**c**) cobalt, and (**d**) chromium. The peaks are resolved into multiple peaks, and the multiplets are numbered.

Overall, the surface of the HEA sample was in the partially oxidized state under the ECM in the neutral nitrate solution. Figure 14a–d show the 2*p*_3/2_ spectra of Fe, Mn, Co, and Cr of the HEA sample potentiostatically conditioned at 5 V in the 20% NaNO_3_ + 0.01 M citric acid solution. The peaks corresponding to unoxidized metallic states (denoted as 1) were larger than the oxidized state peaks for the elements Fe, Mn, and Co. Chromium was predominantly in its oxidized state. These results indicated that adding citric acid to the nitrate solution will help minimize the oxidation of the surface during ECM and will improve the Faradaic efficiency. The Si-*2p* core level energy of 103.8 eV is considered to be associated with SiO_2_, while the binding energy of 99.3 eV is attributed to silicon [50]. In the presence of other oxides, the Si-*2p* core level energies were shifted to lower values. Laurent et al. reported binding energies of 102.1 ± 0.3 eV and 102.7 ± 0.3 eV for Si-*2p*_3/2_ and Si-*2p*_1/2_, respectively [15]. Similar binding energies were recorded for the Fe_42_Mn_28_Cr_15_Co_10_Si_5_ alloy conditioned at lower potentials (0.7 and 1 V), along with a minor peak at 99.0 eV, indicating that the Si was present predominantly as SiO_2_. The samples conditioned at 5 V revealed a broader single peak centered at 100.4 eV, indicating that the silicon was only partially oxidized.

Corrosion and passivation of Fe_50_Mn_30_Co_10_Cr_10_ HEA and their derivatives have been reported by several researchers [51,52]. In this work, the derivative alloy contains silicon in addition to the chromium to enhance the passivation behavior. The XPS results show that the passive film contained all the *3d* transition elements. When nickel is added as an alloying element, the corrosion resistance was observed to be equivalent to that of type 304 SS [53]. On the other hand, the presence of a higher amount of Mn resulted in a higher passivation current density or no passivation in high chloride concentration solutions

### 3.7. Mechanism of Electrochemical Dissolution of the HEA

The potentiostatic ECM experiments revealed that Faradaic efficiency was higher in the 20% NaNO_3_ + citric acid solution and 20% NaCl solution. The experimental results showed that at a high concentration of nitrate, the passivation current was considerably reduced. No passivation was observed in the 20% chloride solution. Therefore, these two electrolytes could be used for the ECM of the Fe_42_Mn_28_Cr_15_Co_10_Si_5_ alloy. The mechanism of the electro-dissolution of iron and other transition metals in nitrate and chloride solutions has been discussed by several research groups [54,55,56,57,58,59,60,61]. Based on the published literature, the electrochemical dissolution mechanism of the Fe_42_Mn_28_Cr_15_Co_10_Si_5_ could be summarized as follows.

M + X^−^ → [MX]_ads_ + e^−^ (6)

[MX]_ads_ → (MX)^+^ + e^−^ (7)

(MX)^+^ → M^2+^ + X^−^ (8)

The overall anodic reaction is:

M → M^2+^ + 2e^−^ (9)

Here, M is the transition metal in the HEA, such as Fe, Mn, Cr, and Co. X indicates an anion in the electrolyte, such as Cl^−^, NO_3_^−^, or OH^−^. The rate-controlling step of the anodic reaction could be either reaction (5) or (6). The anodic current, *I*, associated with reaction (5) can be given as:

I = nFAk_4_a_X_^−^ exp(αFE/RT) (10)

where n = number of electrons, F = Faraday’s constant, A = exposed area of the sample, *k*_4_ = rate constant of reaction (5), a_X_^−^ = activity of the anion, α = transfer coefficient, E = applied potential, R = universal gas constant, and T = temperature.

In addition to the anodic reaction of metal dissolution, other anodic reactions occur, such as oxygen evolution and passivation, which are detrimental to the metal removal process. The cathodic reactions that occur on the counter electrode are water or hydrogen reduction, and reduction of nitrate (in case of the nitrate solution) as given below.

Acid electrolyte:      2H^+^ + 2e^−^ → H_2_      (11)

Neutral electrolyte:   2H_2_O + 2e^−^ → H_2_ + 2OH^−^    (12)

Nitrate reduction:  NO_3_^−^ + 2H^+^ + 2e^−^ → NO_2_^−^ + H_2_O (13)

The overall reaction in the nitrate solution is given as:

3M^2+^ + NO_3_^−^ + 4H^+^ → 3 M^3+^ + NO + 2H_2_O (14)

The above reactions indicate that the pH of the electrolyte would increase during the ECM process. It was observed that the pH of the 20%NaNO_3_ + 0.01 M citric acid increased from 2.16 to 6.22 after the ECM experiment, and the pH of the 20% chloride solution increased from 6 to 10.01.

Electrochemical machining of engineering components is performed by applying a *dc* potential of 10–20 V between the workpiece (anode) and a pre-shaped cathode tool with a current density in the range of 20–200 A/cm^2^. The gap between the anode and cathode is typically maintained in the range of 0.1–0.6 mm [62]. The electrolyte is pumped through the inter-electrode gap at a velocity of 10–60 m/s [25]. The high current density results in a very high dissolution rate. The dissolved ions are carried away from the workpiece surface by the circulating electrolyte, which is pumped at high velocity. The high flow rate also minimizes the concentration polarization and supersaturation/reprecipitation of the dissolved species. The high velocity flow of the electrolyte also minimizes the masking effect of evolving gas bubbles. In this study, experiments were carried out at low current densities in static conditions. Therefore, the dissolution kinetics were limited by the mass transport of the dissolved species. The lower faradaic efficiency observed in this study could be attributed to the static experimental condition. Static electrolytes limit scalability due to poor heat and by-product management, leading to inconsistent machining and surface defects. The future work will involve the application of high current densities (100–200 A/cm^2^) under high velocity electrolyte circulation. The process parameters, such as current density, inter-electrode gap, and flow velocity, will be optimized to achieve a surface roughness (R_a_) of <1 μm.

## 4. Conclusions

The Fe_42_Mn_28_Cr_15_Co_10_Si_5_ alloy comprised ~80% *hcp ε*-martensite and 20% *fcc* austenite in the hot-rolled condition. This material exhibited excellent passivation in 1 M NaNO_3_ solutions with and without acidification using 0.01 M citric or nitric acid. The addition of citric acid resulted in an average passive current density of 15 μA/cm^2^, while the addition of nitric acid showed 35 μA/cm^2^.

The polarization behavior in the higher concentration nitrate solution (20% NaNO_3_ or 2.35 M) with and without the addition of 0.01 M citric or nitric acid showed an order-of-magnitude-higher passivation current density and corrosion current density than that of the 1 M NaNO_3_ counterparts. The HEA samples did not reveal a passivation behavior in the 20% chloride solution.

Electrochemical machining of HEA at constant applied potentials in the range of 5–10 V revealed no preferential attack on particular phases or grain boundaries. Oxygen gas bubbles adhering to the surface affected the surface finish. The nitrate-based solution showed a lower Faradaic efficiency of material removal than the chloride solution. An increase in the potential increased the faradaic efficiency. The maximum efficiency observed in the nitrate-based solution was 21.9%. The reduced efficiency was attributed to the oxygen evolution reaction and passivation.

XPS analysis indicated oxidation of alloying additions during the ECM in neutral nitrate solution. Addition of citric acid minimized the surface oxidation.

The future work will focus on exploring the influence of electrolyte flow rate on the ECM uniformity of HEAs and developing nitrate–chloride composite electrolytes for the ECM of metastable HEAs.

## Figures and Tables

**Table 1:** Electrochemical parameters derived from polarization measurements.

Solution	*E_corr_*^a^, V_Ag/AgCl_	*i_oc_*^b^, A/cm^2^	*i_oa_*^c^, A/cm^2^	*i_corr_*^d^, A/cm^2^	*β_c_*^e^, V/decade	*β*_a_ ^f^, V/decade
20% NaNO_3_	−0.33 ± 0.04	1.30 ± 0.99 × 10^−8^	5.50 ± 3.54 × 10^−6^	2.35 ± 1.77 × 10^−5^	−0.35 ± 0.07	0.59 ± 0.10
20% NaNO_3_ + 10 mM Citric acid	−0.34 ± 0.06	3.02 ± 4.22 × 10^−9^	6.05 ± 7.00 × 10^−6^	3.20 ± 0.14 × 10^−5^	−0.24 ± 0.03	0.64 ± 0.40
20% NaNO_3_ + 10 mM HNO_3_	−0.27 ± 0.01	4.00 ± 5.66 × 10^−15^	4.04 ± 5.60 × 10^−7^	2.70 ± 2.26 × 10^−5^	−0.15 ± 0.10	0.25 ± 0.13
20% NaCl	−0.45 ± 0.04	1.26 ± 1.33 × 10^−8^	1.43 ± 1.94 × 10^−6^	1.45 ± 0.78 × 10^−5^	−0.30 ± 0.00	0.27 ± 0.16
1 M NaNO_3_	−0.255 ± 0.00	1.75 ± 3.50 × 10^−14^	3.00 ± 1.50 × 10^−10^	9.00 ± 3.28 × 10^−7^	−0.14 ± 0.03	0.16 ± 0.13
1 M NaNO_3_ + 0.01 Citric acid	−0.266 ± 0.01	1.20 ± 0.28 × 10^−18^	9.05 ± 4.31 × 10^−11^	2.68 ± 0.40 × 10^−7^	−0.10 ± 0.01	0.14 ± 0.01
1 M NaNO_3_ + 0.01 M HNO_3_	−0.273 ± 0.02	2.00 ± 1.41 × 10^−17^	2.00 ± 1.38 × 10^−9^	1.1 ± 0.51 × 10^−6^	−0.12 ± 0.04	0.20 ± 0.05

^a^ *E_corr_*, Corrosion potential, ^b^ *i_oc_*, cathodic exchange current density, ^c^ *i_oa_*, anodic exchange current density, ^d^ *i_corr_*, corrosion current density, ^e^ *β_c_*, Cathodic tafel slope, ^f^ *β_a_*, Anodic tafel slope.

**Table 2:** Initial pH of the electrolytes and redox potential for oxygen reduction.

Solution	pH	Redox Potential of Oxygen Reduction Reaction, V_Ag/AgCl_
20 wt% NaNO_3_	5.70	0.70
20 wt% NaNO_3_ + 0.01 M HNO_3_,	1.69	0.93
20 wt% NaNO_3_ + 0.01 M Citric acid	2.16	0.90
20 wt% NaCl	5.99	0.68

**Table 3:** Charge accumulated during electrochemical machining at constant potential and faradaic efficiency.

Solution	Applied Potential, V	Total Charge Recorded, A.s	^a^ ∆V_Theoretical_, 10^−3^ cm^3^	^b^ ∆V_measured_, 10^−4^ cm^3^	Faradaic Efficiency, %
20% NaNO_3_	5	109.81 ± 01.19	3.16	2.75 ± 00.53	8.68
	7.5	273.21 ± 10.10	7.85	8.22 ± 00.92	10.45
	10	357.89 ± 42.20	10.30	6.00 ± 01.13	5.83
20% NaNO_3_ + 0.01M HNO_3_	5	111.20 ± 03.08	3.20	3.55 ± 00.93	11.08
	7.5	316.34 ± 10.34	9.09	7.54 ± 01.72	8.30
	10	434.21 ± 24.90	12.50	16.15 ± 02.44	12.90
20% NaNO_3_ + 0.01 M Citric acid	5	98.49 ± 00.79	2.83	1.78 ± 00.20	6.29
	7.5	299.54 ± 02.46	8.61	7.57 ± 00.94	8.78
	10	577.28 ± 39.10	16.60	36.31 ± 04.08	21.9
20% NaCl	5	115.02 ± 11.16	3.30	5.61 ± 00.95	16.94
	7.5	164.64 ± 25.79	4.73	10.10 ± 02.83	21.35
	10	193.43 ± 26.41	5.56	28.45 ± 06.64	57.4

^a^ ∆V_Theoritical_: Theoretical volume loss associated with the charge. ^b^ ∆V_Measured_: Measured volume loss after ECM.

## Data Availability

The original contributions presented in this study are included in the article/Supplementary Materials. Further inquiries can be directed to the corresponding author.

## Supplementary Materials

The following supporting information can be downloaded at: https://www.mdpi.com/article/10.3390/ma18214881/s1, Figure S1: Electrical equivalent circuit model for fitting the EIS data; Table S1: Parameters for the calculation of volume fraction of phases; Table S2: EEC model parameters fitted with the EIS data

## Abbreviations

The following abbreviations are used in this manuscript:

ECM	Electrochemical Machining
EIS	Electrochemical Impedance Spectroscopy
Fcc	Face-centered cubic
Hcp	hexagonally close packed
HEA	High-Entropy Alloy
ORR	Oxygen reduction reaction
PD	Potentiodynamic
PS	Potentiostatic
SEM	Scanning Electron Microscopy
XPS	X-ray photoelectron spectroscopy
XRD	X-ray Diffraction
