# Supplementary material for: Electrochemical Machining of Highly Strain-Hardenable High-Entropy FeMnCrCoSi Alloy: Role of Passivation and Selective Dissolution
Source: Materials (Basel). 2025 Oct 24;18(21):4881. doi: 10.3390/ma18214881 (PMC12608881; doi:10.3390/ma18214881)
Supplement: Supplementary file 1 [file materials-18-04881-s001.zip › materials-3896021-supplementary.pdf]

Supplementary data

Volume fraction Estimation by XRD

The second method considered the highest intensities of the recorded peaks (ε(101) and γ(111)).

The fraction of the phases was calculated using the relation [36]:

$$\frac{c_\varepsilon}{c_\gamma} = \frac{I_\varepsilon R_\gamma}{I_\gamma R_\varepsilon}$$

where c_ε and c_γ are contents of the ε and γ phases, respectively; Iε and Iγ are the recorded integrated intensities; and R is given by the relation:

$$R = F^2 p \left(\frac{1 + cos^2 2\theta}{2Sin^2\theta.cos\theta}\right)\frac{e^{-2M_T}}{\Omega^2}$$

where F = structure factor, p = multiplicity factor, the term in parentheses = Lorentz polarization factor, e^{-2Mt} = temperature factor, and Ω = volume of unit cell. In this calculation, the temperature factor was assumed to be equal in both phases. The calculated values are listed in the Table below:

Table S 1 Parameters for the calculation of volume fraction of phases

Phase	p	LP	F^2	Ω^2 (nm^6)	R	I	Fraction
ε(101)	12	6.01	848.53	5.13 X 10^{-4}	1.19 x 10^8	39690	0.802
γ(111)	8	5.14	4188.67	2.1 x 10^{-3}	8.21 x 10^7	6760	0.198

It is noted that both methods yielded almost similar results (c_ε = 0.79 and 0.8). In this manuscript, the ratio of summed-up integrated intensities of all the recorded peaks (c_ε = 0.79) was reported.

EIS Model

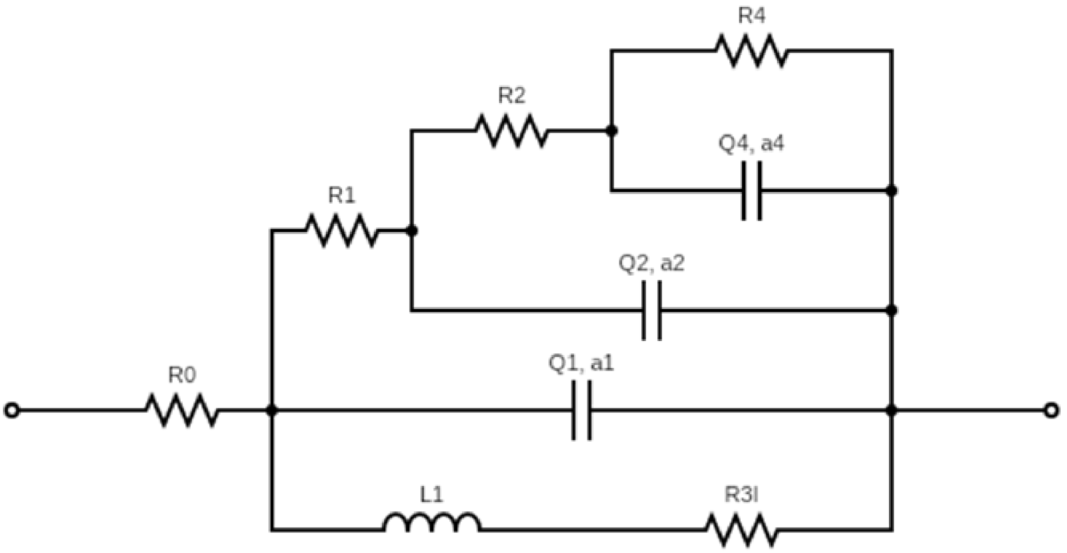

Figure S 1 Electrical equivalent circuit model for fitting the EIS data

Table S 2 EEC model parameters fitted with the EIS data

	20% NaCl	20% $NaNO_3$	20% $NaNO_3$ + 0.01 M Citric	20% $NaNO_3$ + 0.01 M HNO_3
R0 [ohm]	6.62	9.97	10.54	8.53
Q1 [S*s^{a1}]	2.22E-05	8.10E-06	1.18E-05	1.71E-05
a_1	0.78	0.86	0.81	0.82
R_1 [ohm]	3.32E+04	3.02E+02	3.02E+02	1.88E+02
Q2 [S*s^{a2}]	1.23E-04	8.78E-05	1.40E-05	2.98E-05
a_2	0.80	0.71	0.80	0.87
R2 [ohm]	3.04E+04	3.86E+03	6.60E+02	2.76E+02
R4 [ohm]	3.46E+05	6.29E+04	8.28E+04	7.77E+04
Q4 [S*s^{a4}]	7.30E-04	2.47E-06	4.20E-05	6.90E-05
a4	0.68	0.99	0.57	0.51
R3l [ohm]	4.01E+05	3.79E+04	8.23E+04	2.57E+04
L1 [H]	4.59E-07	5.67E-02	6.27E-02	7.18E+00
[Goodness of Fit]	7.54E-04	2.80E-02	1.44E-04	1.62E-04
